# 4-Fluoro-2-[(3-methyl­phen­yl)imino­meth­yl]phenol

**DOI:** 10.1107/S1600536812010513

**Published:** 2012-03-14

**Authors:** Alice Brink, Hendrik G. Visser, Andreas Roodt

**Affiliations:** a Department of Chemistry, University of the Free State, PO Box 339, Bloemfontein 9300, South Africa

## Abstract

The title compound, C_14_H_12_FNO, crystallizes as the *trans* phenol–imine tautomer. The two benzene rings are essentially coplanar, being inclined to one another by 9.28 (7)°. This is at least in part due to the intra­molecular O—H⋯N hydrogen bond between the hy­droxy O atom and the imine N atom. The crystal structure is stabilized by an array of weak C—H⋯O and C—H⋯F inter­actions, which link the mol­ecules into a stable three-dimensional network.

## Related literature

For related structures, see: Karakaş *et al.* (2004 ▶); Arod *et al.* (2005 ▶); Cheng *et al.* (2005 ▶); Brink *et al.* (2009 ▶). For related rhenium tricarbonyl complexes containing salicylaldimines, see: Brink *et al.* (2011 ▶). For related *N,O*-bidentate ligands coordinated to a rhenium tricarbonyl core, see: Schutte *et al.* (2011 ▶).
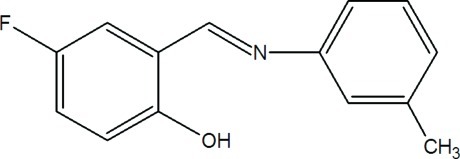


## Experimental

### 

#### Crystal data

C_14_H_12_FNO
*M* *_r_* = 229.25
Monoclinic, 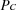

*a* = 10.2655 (6) Å
*b* = 4.6738 (2) Å
*c* = 12.3561 (8) Å
β = 112.331 (3)°
*V* = 548.37 (5) Å^3^
*Z* = 2
Mo *K*α radiation
μ = 0.10 mm^−1^
*T* = 100 K
0.19 × 0.1 × 0.06 mm

#### Data collection

Bruker X8 APEXII 4K Kappa CCD diffractometer
Absorption correction: multi-scan (*SADABS*; Bruker, 2004 ▶) *T*_min_ = 0.981, *T*_max_ = 0.994
7009 measured reflections
1319 independent reflections
1203 reflections with *I* > 2σ(*I*)
*R*_int_ = 0.028

#### Refinement

*R*[*F* ^2^ > 2σ(*F* ^2^)] = 0.034
*wR*(*F* ^2^) = 0.095
*S* = 1.06
1319 reflections
156 parameters
2 restraints
H-atom parameters constrained
Δρ_max_ = 0.19 e Å^−3^
Δρ_min_ = −0.19 e Å^−3^

### 

Data collection: *APEX2* (Bruker, 2005 ▶); cell refinement: *SAINT-Plus* (Bruker, 2004 ▶); data reduction: *SAINT-Plus* and *XPREP* (Bruker, 2004 ▶); program(s) used to solve structure: *SIR92* (Altomare *et al.*, 1999 ▶); program(s) used to refine structure: *SHELXL97* (Sheldrick, 2008 ▶); molecular graphics: *DIAMOND* (Brandenburg & Putz, 2004 ▶); software used to prepare material for publication: WingGX (Farrugia, 1999 ▶).

## Supplementary Material

Crystal structure: contains datablock(s) global, I. DOI: 10.1107/S1600536812010513/sj5208sup1.cif

Structure factors: contains datablock(s) I. DOI: 10.1107/S1600536812010513/sj5208Isup2.hkl

Supplementary material file. DOI: 10.1107/S1600536812010513/sj5208Isup3.cml

Additional supplementary materials:  crystallographic information; 3D view; checkCIF report

## Figures and Tables

**Table 1:** Hydrogen-bond geometry (Å, °)

*D*—H⋯*A*	*D*—H	H⋯*A*	*D*⋯*A*	*D*—H⋯*A*
O1—H1*B*⋯N1	0.84	1.85	2.601 (2)	147
C1—H1*A*⋯O1^i^	0.95	2.6	3.467 (3)	151
C16—H16⋯O1^i^	0.95	2.65	3.495 (3)	149
C13—H13⋯F1^ii^	0.95	2.6	3.472 (3)	153
C231—H23*A*⋯F1^iii^	0.98	2.73	3.321 (3)	119
C231—H23*C*⋯F1^iv^	0.98	2.67	3.193 (2)	114

Symmetry codes: (i) 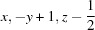; (ii) 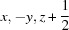; (iii) 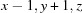; (iv) 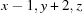.

**Fig. 1.**
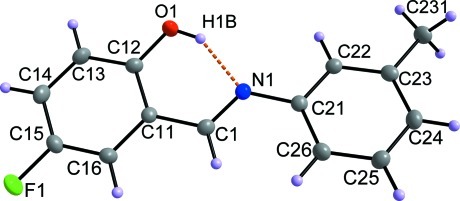
Representation of the molecular structure of the title compound, showing the numbering scheme and displacement ellipsoids drawn at the 50% probability level.

**Fig. 2.**
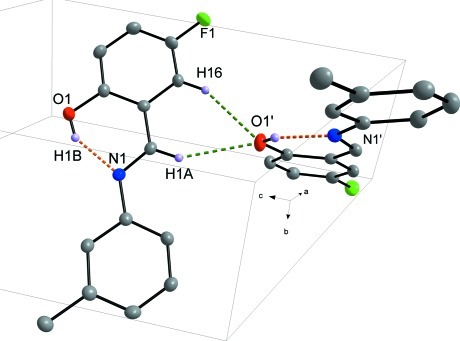
Representation of the hydrogen-bond interactions (only relevant H atoms are shown).

**Fig. 3.**
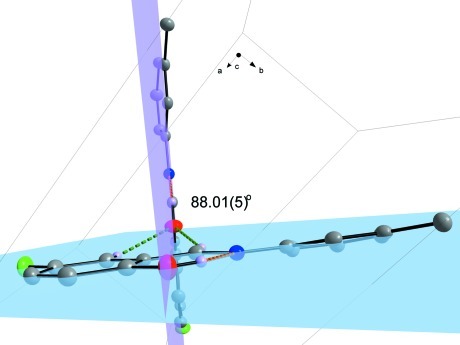
Representation of the perpendicular orientation of molecules.

**Fig. 4.**
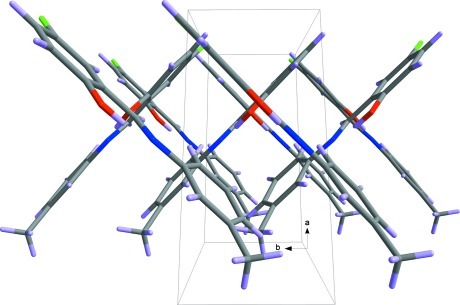
Molecular packing of the unit cell illustrating the cube-like formation as viewed along the c-axis.

## supplementary crystallographic information

### Comment

Schiff-base ligands have played a significant role in the development of coordination chemistry as stable organometallic complexes are readily formed with a variety of transition metals. In continuation of our research on the coordination of various bifunctional chelate systems on the *fac*-[*M*(CO)_3_]^+^ moiety (*M* = Re(I), Tc(I)) (Brink *et al.*, 2011; Schutte *et al.*, 2011) the title compound was synthesized and is reported here.

The title compound (Figure 1) is essentially co-planar with a dihedral angle of 9.28 (7)° between the aromatic rings. The bond distances and angles in the title compound are in accord with those reported for related salicylaldimine-based ligand systems (Karakaş *et al.*, 2004; Arod *et al.*, 2005; Cheng *et al.*, 2005; Brink *et al.*, 2009).

The compound crystallizes as the *trans* phenol-imine tautomer. A strong intramolecular hydrogen bond occurs between the O—H···N atoms in each unique molecule. The crystal structure is stabilized by an array of weak C—H···O and C—H···F interactions. The bifurcated acceptor, O1, experiences weak hydrogen bond interactions with H16 and H1A. As a result, the two independent molecules pack nearly perpendicular to each other with a dihedral angle of 88.01 (5)° between planes drawn through the C1 aromatic ring systems (Figures 2 and 3). All the interactions serve to link the molecules into a stable three-dimensional supramolecular network. The molecular packing, viewed along the *c*-axis, illustrates the cube-like tunnel formation resulting from the various interactions (Figure 4).

### Experimental

The reaction was performed under Schlenk conditions using a nitrogen atmosphere. To a solution of 5-fluorosalicylaldehyde (0.50 g, 3.57 mmol) in methanol, a solution of *m*-toluidine (0.382 g, 3.57 mmol) was added. The reaction was refluxed at 80°C for 3 h. The solvent was removed under reduced pressure. The product was obtained as an orange solid which was washed with cold methanol and filtered. Crystals suitable for X-ray diffraction were grown from the filtrate. Yield 82.1%. ^1^H NMR [acetone-*d_6_*, 600 MHz, δ (p.p.m.)] 13.04 (s, 1H), 8.90 (s, 1H), 7.40 (dd, 1H, *J* = 3.1, 8.7 Hz), 7.35 (t, 1H, *J* = 7.7 Hz), 7.25 (s, 1H), 7.24–7.20 (m, 2H), 7.16 (d, 1H, *J* = 7.7 Hz), 6.96 (dd, 1H, *J* = 4.5, 9.1 Hz), 2.39 (s, 3H, CH_3_).

### Refinement

The aromatic H atoms and hydroxy H atom were placed in geometrically idealized positions and constrained to ride on their parent atoms with *U*_iso_(H) = 1.2*U*_eq_(C) and 1.5_eq_(O).The aliphatic H atoms were placed in geometrically idealized positions and constrained to ride on their parent atoms with *U*_iso_(H) = 1.2*U*_eq_(C) and 1.5_eq_(C), respectively for the methylene and methyl carbon atoms. The methyl groups were generated to fit the difference electron density and the groups were then refined as rigid rotors. The absolute structure parameter is meaningless and has been removed from the CIF. The Friedel opposites have been merged as the compound is a weak anomalous scatterer.

### Crystal data

**Table:**

C_14_H_12_FNO	*F*(000) = 240
*M**_r_* = 229.25	*D*_x_ = 1.388 Mg m^−^^3^
Monoclinic, *P**c*	Mo *K*α radiation, λ = 0.71073 Å
Hall symbol: P -2yc	Cell parameters from 2573 reflections
*a* = 10.2655 (6) Å	θ = 3.4–28.3°
*b* = 4.6738 (2) Å	µ = 0.10 mm^−^^1^
*c* = 12.3561 (8) Å	*T* = 100 K
β = 112.331 (3)°	Cuboid, orange
*V* = 548.37 (5) Å^3^	0.19 × 0.1 × 0.06 mm
*Z* = 2	

### Data collection

**Table:**

Bruker X8 APEXII 4K Kappa CCD diffractometer	1319 independent reflections
Graphite monochromator	1203 reflections with *I* > 2σ(*I*)
Detector resolution: 512 pixels mm^-1^	*R*_int_ = 0.028
ω and φ scans	θ_max_ = 28.0°, θ_min_ = 3.4°
Absorption correction: multi-scan (*SADABS*; Bruker, 2004)	*h* = −13→13
*T*_min_ = 0.981, *T*_max_ = 0.994	*k* = −5→6
7009 measured reflections	*l* = −16→16

### Refinement

**Table:**

Refinement on *F*^2^	2 restraints
Least-squares matrix: full	H-atom parameters constrained
*R*[*F*^2^ > 2σ(*F*^2^)] = 0.034	*w* = 1/[σ^2^(*F*_o_^2^) + (0.0597*P*)^2^ + 0.0513*P*] where *P* = (*F*_o_^2^ + 2*F*_c_^2^)/3
*wR*(*F*^2^) = 0.095	(Δ/σ)_max_ < 0.001
*S* = 1.06	Δρ_max_ = 0.19 e Å^−^^3^
1319 reflections	Δρ_min_ = −0.19 e Å^−^^3^
156 parameters	

### Special details

**Table:**

Experimental. Intensity data was collected on a Bruker X8 Apex II 4 K Kappa CCD diffractometer using an exposure time of 55 s/frame. A total of 1495 frames were collected with a frame width of 0.5° covering up to θ = 28.0° with 99.6% completeness accomplished
Geometry. All e.s.d.'s (except the e.s.d. in the dihedral angle between two l.s. planes) are estimated using the full covariance matrix. The cell e.s.d.'s are taken into account individually in the estimation of e.s.d.'s in distances, angles and torsion angles; correlations between e.s.d.'s in cell parameters are only used when they are defined by crystal symmetry. An approximate (isotropic) treatment of cell e.s.d.'s is used for estimating e.s.d.'s involving l.s. planes.

### Fractional atomic coordinates and isotropic or equivalent isotropic displacement parameters (Å^2^)

**Table:**

	*x*	*y*	*z*	*U*_iso_*/*U*_eq_	
N1	0.51676 (18)	0.7931 (4)	0.51611 (15)	0.0170 (4)	
O1	0.62483 (16)	0.5330 (3)	0.71672 (14)	0.0229 (4)	
H1B	0.571	0.6442	0.666	0.034*	
F1	0.95777 (14)	−0.0665 (3)	0.54083 (13)	0.0276 (3)	
C1	0.5962 (2)	0.6410 (4)	0.47953 (19)	0.0178 (4)	
H1A	0.5897	0.6626	0.4012	0.021*	
C11	0.6955 (2)	0.4372 (4)	0.55563 (18)	0.0161 (4)	
C12	0.7069 (2)	0.3900 (4)	0.67131 (18)	0.0183 (4)	
C13	0.8048 (2)	0.1934 (5)	0.74184 (18)	0.0211 (5)	
H13	0.8132	0.1637	0.8203	0.025*	
C14	0.8897 (2)	0.0416 (5)	0.6980 (2)	0.0219 (5)	
H14	0.9567	−0.0919	0.7458	0.026*	
C15	0.8756 (2)	0.0873 (4)	0.5839 (2)	0.0195 (5)	
C16	0.7817 (2)	0.2812 (5)	0.51227 (19)	0.0181 (4)	
H16	0.7753	0.3092	0.4343	0.022*	
C21	0.4220 (2)	0.9970 (4)	0.44138 (18)	0.0168 (4)	
C22	0.3265 (2)	1.1211 (4)	0.48298 (18)	0.0171 (4)	
H22	0.3286	1.0669	0.5577	0.021*	
C23	0.2280 (2)	1.3232 (5)	0.41733 (18)	0.0189 (4)	
C24	0.2290 (2)	1.4038 (5)	0.30877 (18)	0.0209 (5)	
H24	0.163	1.541	0.2625	0.025*	
C25	0.3257 (2)	1.2853 (5)	0.26779 (19)	0.0223 (5)	
H25	0.3258	1.3445	0.1943	0.027*	
C26	0.4219 (2)	1.0818 (4)	0.33261 (19)	0.0205 (5)	
H26	0.4871	1.0008	0.3036	0.025*	
C231	0.1250 (2)	1.4526 (5)	0.4626 (2)	0.0227 (5)	
H23A	0.1367	1.3641	0.5377	0.034*	
H23B	0.0289	1.4194	0.4063	0.034*	
H23C	0.1422	1.6588	0.4736	0.034*	

### Atomic displacement parameters (Å^2^)

**Table:**

	*U*^11^	*U*^22^	*U*^33^	*U*^12^	*U*^13^	*U*^23^
N1	0.0165 (8)	0.0168 (8)	0.0174 (9)	−0.0004 (6)	0.0061 (7)	0.0005 (6)
O1	0.0264 (8)	0.0271 (8)	0.0181 (7)	0.0081 (6)	0.0116 (7)	0.0032 (6)
F1	0.0278 (7)	0.0264 (7)	0.0320 (7)	0.0092 (5)	0.0154 (6)	0.0010 (6)
C1	0.0200 (10)	0.0174 (8)	0.0165 (10)	−0.0022 (8)	0.0075 (8)	0.0011 (8)
C11	0.0166 (10)	0.0141 (9)	0.0164 (10)	−0.0015 (7)	0.0050 (8)	−0.0008 (8)
C12	0.0190 (10)	0.0193 (10)	0.0170 (10)	−0.0024 (8)	0.0074 (9)	−0.0012 (8)
C13	0.0228 (11)	0.0231 (10)	0.0157 (10)	−0.0005 (9)	0.0053 (9)	0.0034 (8)
C14	0.0201 (11)	0.0207 (10)	0.0212 (11)	0.0009 (8)	0.0036 (9)	0.0019 (8)
C15	0.0166 (10)	0.0191 (10)	0.0244 (12)	0.0003 (8)	0.0096 (9)	−0.0018 (8)
C16	0.0194 (10)	0.0192 (10)	0.0178 (10)	−0.0010 (8)	0.0095 (8)	−0.0006 (7)
C21	0.0171 (10)	0.0154 (9)	0.0177 (10)	−0.0020 (8)	0.0063 (8)	−0.0010 (7)
C22	0.0184 (10)	0.0163 (9)	0.0165 (10)	−0.0023 (7)	0.0066 (8)	−0.0006 (8)
C23	0.0168 (10)	0.0188 (10)	0.0200 (11)	−0.0025 (8)	0.0059 (8)	−0.0034 (8)
C24	0.0181 (11)	0.0193 (10)	0.0219 (11)	0.0017 (8)	0.0039 (9)	0.0011 (8)
C25	0.0240 (12)	0.0248 (11)	0.0186 (10)	0.0027 (8)	0.0086 (9)	0.0038 (8)
C26	0.0206 (10)	0.0217 (10)	0.0209 (11)	0.0022 (8)	0.0098 (9)	0.0001 (8)
C231	0.0192 (10)	0.0244 (10)	0.0247 (11)	0.0015 (8)	0.0086 (9)	−0.0019 (9)

### Geometric parameters (Å, º)

**Table:**

N1—C1	1.287 (3)	C21—C22	1.395 (3)
N1—C21	1.422 (3)	C21—C26	1.401 (3)
O1—C12	1.353 (3)	C21—N1	1.422 (3)
O1—H1B	0.84	C22—C23	1.396 (3)
F1—C15	1.361 (2)	C22—H22	0.95
C1—C11	1.450 (3)	C23—C24	1.397 (3)
C1—H1A	0.95	C23—C231	1.499 (3)
C11—C16	1.401 (3)	C24—C25	1.389 (3)
C11—C12	1.407 (3)	C24—H24	0.95
C12—C13	1.396 (3)	C25—C26	1.386 (3)
C13—C14	1.385 (3)	C25—H25	0.95
C13—H13	0.95	C26—H26	0.95
C14—C15	1.377 (3)	C231—H23A	0.98
C14—H14	0.95	C231—H23B	0.98
C15—C16	1.373 (3)	C231—H23C	0.98
C16—H16	0.95		
			
C1—N1—C21	120.69 (16)	C26—C21—N1	124.30 (18)
C12—O1—H1B	109.5	C22—C21—N1	116.25 (17)
N1—C1—C11	121.23 (18)	C26—C21—N1	124.30 (18)
N1—C1—H1A	119.4	C21—C22—C23	121.53 (18)
C11—C1—H1A	119.4	C21—C22—H22	119.2
C16—C11—C12	119.11 (19)	C23—C22—H22	119.2
C16—C11—C1	118.98 (18)	C22—C23—C24	118.16 (18)
C12—C11—C1	121.91 (18)	C22—C23—C231	120.94 (18)
O1—C12—C13	118.70 (18)	C24—C23—C231	120.90 (19)
O1—C12—C11	121.31 (19)	C25—C24—C23	120.63 (19)
C13—C12—C11	119.99 (19)	C25—C24—H24	119.7
C14—C13—C12	120.27 (19)	C23—C24—H24	119.7
C14—C13—H13	119.9	C26—C25—C24	120.94 (19)
C12—C13—H13	119.9	C26—C25—H25	119.5
C15—C14—C13	118.9 (2)	C24—C25—H25	119.5
C15—C14—H14	120.5	C25—C26—C21	119.3 (2)
C13—C14—H14	120.5	C25—C26—H26	120.4
F1—C15—C16	118.91 (19)	C21—C26—H26	120.4
F1—C15—C14	118.57 (19)	C23—C231—H23A	109.5
C16—C15—C14	122.5 (2)	C23—C231—H23B	109.5
C15—C16—C11	119.17 (19)	H23A—C231—H23B	109.5
C15—C16—H16	120.4	C23—C231—H23C	109.5
C11—C16—H16	120.4	H23A—C231—H23C	109.5
C22—C21—C26	119.44 (19)	H23B—C231—H23C	109.5
C22—C21—N1	116.25 (17)		
			
N1—N1—C1—C11	0.0 (6)	N1—N1—C21—C22	0.0 (6)
C21—N1—C1—C11	178.58 (17)	C1—N1—C21—C22	171.08 (18)
N1—C1—C11—C16	−178.66 (18)	N1—N1—C21—C26	0.0 (7)
N1—C1—C11—C12	1.9 (3)	C1—N1—C21—C26	−10.4 (3)
C16—C11—C12—O1	−179.24 (18)	C1—N1—C21—N1	0E1 (10)
C1—C11—C12—O1	0.2 (3)	C26—C21—C22—C23	1.7 (3)
C16—C11—C12—C13	1.1 (3)	N1—C21—C22—C23	−179.74 (17)
C1—C11—C12—C13	−179.45 (19)	N1—C21—C22—C23	−179.74 (17)
O1—C12—C13—C14	179.49 (19)	C21—C22—C23—C24	−1.3 (3)
C11—C12—C13—C14	−0.8 (3)	C21—C22—C23—C231	179.38 (19)
C12—C13—C14—C15	−0.2 (3)	C22—C23—C24—C25	0.0 (3)
C13—C14—C15—F1	−178.87 (18)	C231—C23—C24—C25	179.33 (19)
C13—C14—C15—C16	1.0 (3)	C23—C24—C25—C26	0.9 (3)
F1—C15—C16—C11	179.14 (17)	C24—C25—C26—C21	−0.5 (3)
C14—C15—C16—C11	−0.8 (3)	C22—C21—C26—C25	−0.7 (3)
C12—C11—C16—C15	−0.3 (3)	N1—C21—C26—C25	−179.19 (19)
C1—C11—C16—C15	−179.79 (18)	N1—C21—C26—C25	−179.19 (19)

### Hydrogen-bond geometry (Å, º)

**Table:**

*D*—H···*A*	*D*—H	H···*A*	*D*···*A*	*D*—H···*A*
O1—H1*B*···N1	0.84	1.85	2.601 (2)	147
C1—H1*A*···O1^i^	0.95	2.6	3.467 (3)	151
C16—H16···O1^i^	0.95	2.65	3.495 (3)	149
C13—H13···F1^ii^	0.95	2.6	3.472 (3)	153
C231—H23*A*···F1^iii^	0.98	2.73	3.321 (3)	119
C231—H23*C*···F1^iv^	0.98	2.67	3.193 (2)	114

Symmetry codes: (i) *x*, −*y*+1, *z*−1/2; (ii) *x*, −*y*, *z*+1/2; (iii) *x*−1, *y*+1, *z*; (iv) *x*−1, *y*+2, *z*.
